# α-Actinin-4 Promotes the Progression of Prostate Cancer Through the Akt/GSK-3β/β-Catenin Signaling Pathway

**DOI:** 10.3389/fcell.2020.588544

**Published:** 2020-12-10

**Authors:** Sungyeon Park, Minsoo Kang, Suhyun Kim, Hyoung-Tae An, Jan Gettemans, Jesang Ko

**Affiliations:** ^1^Division of Life Sciences, Korea University, Seoul, South Korea; ^2^Department of Biomolecular Medicine, Ghent University, Ghent, Belgium

**Keywords:** actinin-4, androgen-independent prostate cancer, AIPC transition, β-catenin, nanobody

## Abstract

The first-line treatment for prostate cancer (PCa) is androgen ablation therapy. However, prostate tumors generally recur and progress to androgen-independent PCa (AIPC) within 2–3 years. α-Actinin-4 (ACTN4) is an actin-binding protein that belongs to the spectrin gene superfamily and acts as an oncogene in various cancer types. Although ACTN4 is involved in tumorigenesis and the epithelial–mesenchymal transition of cervical cancer, the role of ACTN4 in PCa remains unknown. We found that the ACTN4 expression level increased during the transition from androgen-dependent PCa to AIPC. ACTN4 overexpression resulted in enhanced proliferation and motility of PCa cells. Increased β-catenin due to ACTN4 promoted the transcription of genes involved in proliferation and metastasis such as *CCND1* and *ZEB1*. ACTN4-overexpressing androgen-sensitive PCa cells were able to grow in charcoal-stripped media. In contrast, ACTN4 knockdown using si-ACTN4 and ACTN4 nanobody suppressed the proliferation, migration, and invasion of AIPC cells. Results of the xenograft experiment revealed that the mice injected with LNCaP^ACTN4^ cells exhibited an increase in tumor mass compared with those injected with LNCaP^Mock^ cells. These results indicate that ACTN4 is involved in AIPC transition and promotes the progression of PCa.

## Introduction

Prostate cancer (PCa) is the most commonly diagnosed cancer, which accounts for 42% of all cancer cases in the United States (Siegel et al., 2017). The androgen receptor (AR) plays an important role in the development of early stage PCa. Upon binding of an androgen, AR is translocated into the nucleus and binds to the androgen receptor elements (AREs) in the promoters of target genes involved in cell proliferation and survival (Tan et al., 2015). Therefore, androgen deprivation therapy is the first-line treatment of PCa patients with local or metastatic prostate tumors; however, tumors relapse to AIPC or castration-resistant PCa (CRPC) within 2–3 years (Widmark et al., 2009; Warde et al., 2011). The progression of CRPC is very aggressive and metastatic (Katsogiannou et al., 2015). Therefore, studies investigating the mechanism underlying the emergence of CRPC have received increasing attention. The following four hypotheses have been proposed to explain the mechanism underlying CRPC development: (1) increased sensitivity of the AR to its agonists; (2) AR mutations activated by non-androgen ligands such as estrogen, progesterone, and glucocorticoids; (3) ligand-independent AR activation through Akt, HER2, and Ack1 kinases; and (4) AR-independent mechanisms (Feldman and Feldman, 2001; Harris et al., 2009; Green et al., 2012; Sharifi, 2013; Tan et al., 2015). However, the exact mechanism underlying CRPC transition and progression has not been elucidated to date.

The PI3K/Akt pathway is a signaling pathway that promotes cell survival and growth in response to various signals. The GSK-3β/β-catenin signaling also plays a role in cell proliferation. Reportedly, the phosphorylation of Akt at Ser9 inhibits GSK-3β activity (Ge et al., 2018). The inhibition of GSK-3β induces β-catenin stabilization in the cytosol; subsequently, the accumulated β-catenin is translocated into the nucleus and participates in target gene expression (Zeng et al., 1997). Aberrant β-catenin pathway plays a pivotal role in various cancers (Kypta and Waxman, 2012; Asem et al., 2016). High β-catenin levels increase the transcription of genes involved in cell proliferation including *CCND1*, *c-myc*, and *c-jun* (Tetsu and McCormick, 1999). β-Catenin regulates the epithelial–mesenchymal transition (EMT)-related factors, such as E-cadherin and ZEB1, which participate in tumor growth, cell motility, maintenance of cancer stem cell properties, and drug resistance (Nauseef and Henry, 2011; Sanchez-Tillo et al., 2011; Khan et al., 2015; Hanrahan et al., 2017; Montanari et al., 2017; Brabletz et al., 2018).

α-Actinin (ACTN) is an actin-binding cytoskeletal protein. In humans, ACTN has the following four types of isoforms: ACTN1, 2, 3, and 4 (Honda et al., 1998). These are classified into two categories: muscle ACTN2 and 3 and non-muscle ACTN1 and 4 (Millake et al., 1989; Youssoufian et al., 1990). ACTN4 is abundant in various cancers such as pancreatic, cervical, and melanoma cancers, and it is a known oncogene (Honda et al., 1998, 2005; Honda, 2015). ACTN4 functions as a transcriptional co-activator of NF-κB by binding to a NF-κB subunit (Aksenova et al., 2013). The knockdown of ACTN4 inhibits Akt phosphorylation, resulting in the suppression of cell proliferation (Ding et al., 2006). We have previously reported that ACTN4 maintains β-catenin stability by Akt activation to promote EMT and tumorigenesis in cervical cancer (An et al., 2016). However, the role of ACTN4 in PCa remains obscure. In this study, we found that ACTN4 increases cell proliferation and motility in androgen-dependent PCa and AIPC. In addition, we demonstrated that ACTN4 induces tumor growth after castration *in vivo*.

## Materials and Methods

### Materials

Roswell Park Memorial Institute (RPMI) 1640 medium was purchased from Thermo Fisher Scientific (Waltham, MA, United States), and fetal bovine serum (FBS) was obtained from HyClone Laboratories (Logan, UT, United States). Penicillin and streptomycin were purchased from Invitrogen (Carlsbad, CA, United States). G-418 was obtained from LPS solution (Daejeon, South Korea). Antibodies for ACTN4, cyclin D1, and β-actin were purchased from Santa Cruz Biotechnology (Santa Cruz, CA, United States). Anti-ZEB1 and anti-β-catenin (Ser33/37/Thr41) antibodies were obtained from Cell Signaling Technology (Boston, MA, United States). Charcoal was purchased from Millipore Sigma (Burlington, MA, United States).

### Cell Culture

LNCaP, PC3, and DU145 cells were maintained in RPMI 1640 medium supplemented with 10% FBS and 1% penicillin/streptomycin at 37°C in a CO_2_ incubator. LNCaP-AI cells were generated from LNCaP cells by incubation for more than 3 months in RPMI 1640 medium supplemented with 10% charcoal-stripped serum media (CSS) and 1% penicillin/streptomycin at 37°C in a CO_2_ incubator. The two different clonal isolates of LNCaP-AI cells were generated independently and numbered #1 and #2.

### Transfection and Western Blotting

For transfection, human ACTN4 was cloned from human genomic DNA and inserted into pCMV-3tag-1 vector (Agilent Technologies, Santa Clara, CA, United States) using *Hin*dIII and *Xho*I restriction enzymes. Cells were plated at a density of 4 × 10^5^ cells/well. Cells were transfected with plasmids using E-fection plus (LugenSci, Seoul, South Korea) and incubated for 24 h. LNCaP cells were transfected with Flag-Mock or Flag-ACTN4 (1 μg) in RPMI 1640 medium. For si-ACTN4 transfection, cells were transfected with si-ACTN4 using interferin (Polyplus, New York, NY, United States). The sequence of si-ACTN4 was 5′-GACCAGAGAGCUUGAGUATTUACUCAAUCA-GCUCUGGUCTT-3′. For western blotting, cells were lysed using RIPA buffer and centrifuged at 12,000 × *g* for 20 min at 4°C. Equal amount of proteins was electrophoresed on a 10% SDS-PAGE and transferred to nitrocellulose membranes. The membranes were probed with the specific antibodies at 4°C and incubated overnight. β-Actin was used as an internal control. The blots were then incubated with the secondary antibody at 25°C for 1 h. The immune complex was detected using West Save Gold (Young In Frontier, Seoul, South Korea).

### RNA Extraction and RT-PCR

Total RNA was isolated using the TaKaRa MiniBest Universal RNA Extraction Kit (Takara Bio, Kusatsu, Japan) according to the manufacturer’s protocol. cDNA was synthesized from total RNA using 5 × PrimeScript RT master mix (Takara Bio). Quantitative RT-PCR (qRT-PCR) was performed on Quantstudio3 (Thermo Fisher Scientific, Waltham, MA, United States) using EvaGreen 2 × master mix (abm, Vancouver, BC, Canada). Semi-qRT-PCR was performed as previously described (Kang et al., 2011). The primer sequences used in this study are listed in Table 1.

**TABLE 1:** The primer sequences used for RT-PCR.

**(1) qRT-PCR**		
*ACTN4*	Forward	5′-GAACGACCGGCAGGGTGAGG-3′
	Reverse	5′-TCGGTGGTCTCCCGCGACAT-3′
*SNAI1*	Forward	5′-ACCACTATGCCGCGCTCTT-3′
	Reverse	5′-GGTCGTAGGGCTGCTGGAA-3′
*CCND1*	Forward	5′-TGTTGCAGTGAGGGCAAGAA-3′
	Reverse	5′-GACCCTGGTTGCTTCAAGGA-3′
*ZEB1*	Forward	5′-TTCAAACCCATAGTGGTTGCT-3′
	Reverse	5′-TGGGAGATACCAAACCAACTG-3′
*CDH1*	Forward	5′-AACAACGAGATTCTACAAGCCTC-3′
	Reverse	5′-TCGCGTTCCTCCAGTTTTCTT-3′
*CDH2*	Forward	5′-CATCCCTCCAATCAACTTGCC-3′
	Reverse	5′-GAGGCTGGTCAGCTCCTG-3′
*VIM*	Forward	5′-TCTACCAGGTCCTCCAGAGC-3′
	Reverse	5′-CTCCATCCTCCAGACCGAGA-3′
β*-actin*	Forward	5′-TTCTACAATGAGCTGCGTGTG-3′
	Reverse	5′-GGGGTGTTGAAGGTCTCAAA-3′
**(2) Semi-qRT-PCR**		
*ACTN4*	Forward	5′-GGGCAGAAGAGATTGTGGAC-3′
	Reverse	5′-TTGTTCAGGTTGGTGACAGG-3′
*AR*	Forward	5′-CAGGCAGAAGACATCTGAAG-3′
	Reverse	5′-CTCACCAAGCTCCTGGACTC-3′
*KLK3 (PSA)*	Forward	5′-GGCAGGTGCTTGTAGCCTCTC-3′
	Reverse	5′-CACCCGAGCAGGTGCTTTTGC-3′
*GAPDH*	Forward	5′-CCATCACCATCTTCCAGGAG-3′
	Reverse	5′-CCTGCTTCACCACGTTCTTG-3′

### Wound Healing Assay

Stable LNCaP cells were grown to confluence in six-well culture plates and pretreated with mitomycin C (10 μg/ml) for 2 h. An artificial “wound” was created using culture-inserts two well (ibidi, Martinsried, Germany). Cell migration into the wounded region was visualized using an Axiovert 100 fluorescent microscope (Carl Zeiss, Jena, Germany) and images were acquired at the indicated time points.

### Cell Proliferation and Colony Formation Assays

LNCaP or LNCaP-AI cells were transfected with Flag-ACTN4 or si-ACTN4 and seeded onto 96-well plates at a density of 2 × 10^3^ cells/well. Cell proliferation assay was performed using EZ-cytox (DoGenBio, Gyeonggi, South Korea) according to the manufacturer’s protocol. For the colony formation assay, LNCaP or LNCaP-AI cells were transfected with Flag-ACTN4 or si-ACTN4, seeded onto 12-well plates at a density of 3 × 10^3^ cells/well, and incubated for 10 days. Colonies were fixed with 4% paraformaldehyde for 30 min at 25°C and stained using 0.05% crystal violet (Millipore Sigma).

### Cell Migration and Invasion Assays

LNCaP cells (1 × 10^5^) in serum-free media were added to the top chambers of 24-well transwell plates (8 μm pore size; BD Biosciences, San Diego, CA, United States). Complete media were added to the bottom chambers for 48 h. LNCaP-AI cells were transfected with si-ACTN4 and 1 × 10^5^ cells in serum-free media were added to the top chambers of 24-well transwell plates. For the Matrigel invasion assay, transwell inserts were coated with 200 μg/ml Matrigel (Millipore Sigma) diluted with serum-free RPMI 1640 medium and allowed to solidify for 5 h. After harvesting, the cells were suspended in serum-free RPMI 1640 medium at a density of 1.5 × 10^5^ cells/100 μl, and the cell suspension was transferred immediately into the upper compartment of the plate. Subsequently, the lower compartment was filled with complete medium. After 72 h of incubation, the non-invading cells on the upper surface of the membrane were removed by wiping with cotton-tipped swabs. Cells on the lower surface of the membrane were stained with 0.05% crystal violet according to the manufacturer’s protocol. Cell counts of the adherent cells were obtained for five randomly selected fields per well and values were expressed as the average of the counts.

### Cellular Fractionation Assay

Cells were lysed with cytosolic lysis buffer (10 mM NaCl HEPES). Cell lysates were centrifuged at 2,000 × *g* for 10 min at 4°C. Supernatants were used as the cytosolic fraction. Pellets were washed four times using cytosolic lysis buffer and lysed with nuclear lysis buffer (0.4 M NaCl HEPES). Lysates were centrifuged at 12,000 × *g* for 20 min at 4°C. Supernatants were used as the nuclear fraction. Each fraction was subjected to western blot analysis. α-Tubulin was used as an internal control for the cytosolic fraction, and Lamin A/C was used as an internal control for the nuclear fraction.

### Fluorescence Microscopic Analysis

Cells were plated on confocal dishes (SPL Life Sciences, Gyeonggi, South Korea) at a density of 5 × 10^4^ cells/well. After 24 h, the cells were fixed with 4% paraformaldehyde for 10 min and permeabilized with 0.2% Triton X-100 for 5 min. The cells were then incubated with 1% bovine serum albumin at 4°C for 1 h and incubated with specific antibodies at 25°C for 1 h. The cells were incubated with 1 μg/ml Alexa 594 and Alexa 488 (Life Technologies) at 25°C for 30 min. After washing with PBS, the cells were incubated with 200 ng/ml DAPI (Millipore Sigma) at 37°C for 5 min and washed twice with PBS. Fluorescence intensity was captured using the LSM 700 confocal laser scanning microscope (Carl Zeiss, Jena, Germany) (original magnification, × 40).

### Chromatin Immunoprecipitation

Cells were treated with formaldehyde (Millipore Sigma) and rotated at 25°C for 15 min. Glycine (Duchefa Biochemie, Haarlem, Netherlands) was added before scraping with cold PBS. Pellets were lysed by sonication with FA lysis buffer. Chromatin samples were co-immunoprecipitated with protein A/G beads and 5 mg of anti-IgG and anti-β-catenin antibodies. Samples were treated with proteinase K (5 mg/ml) at 65°C for 6 h, followed by phenol/chloroform extraction. Pellets were dissolved in 30 ml of distilled deionized water. For the TCF binding site at position −578 of the human ZEB1 promoter, the primers amplifying the region between −674 and −477 were as follows: forward, 5′-TGGAAGGGAAGGGAAGGGAGTC-3′ and reverse, 5′-AGGCAGGGCTACCATCAGTC-3′. For the TCF binding site at position −161 of the human ZEB1 promoter, the primers amplifying the region between −325 and −101 were as follows: forward, 5′-TTTACCTTTCCAACTCCGACAGC-3′ and reverse, 5′-GGCTTTACGACATCACCTTCCTTAC-3′.

### Animal Study

Six weeks old male BALB/c nude mice were purchased from Orient Bio Inc. (Seongnam, South Korea). Mice were maintained at 22 ± 2°C and 50 ± 10% humidity under a 12 h light:12 h dark regimen. The Institutional Animal Care and Use Committee of Korea University approved the studies, which were performed under the guidelines for the care and use of laboratory animals. LNCaP^Mock^ (4 × 10^6^ cells/100 μl PBS) and LNCaP^ACTN4#2^ (3 × 10^6^ cells/100 μl PBS) cells were subcutaneously injected into mice (*n* = 3). Tumor volume was measured once a week using a digital caliper and calculated according to the following equation: *V* = (width^2^ × length) × 0.5. When the tumor volume reached 400 mm^3^, mice were surgically castrated and monitored for tumor progression.

### Statistical Analysis

Data are presented as the mean ± SEM. Statistical evaluation was performed using GraphPad Prism 5 software (GraphPad Software, La Jolla, CA, United States). Two-tailed Student’s *t*-test was used to determine the significant difference between the means of two independent groups. Two-tailed Student’s *t*-test values of *p* < 0.05 were considered statistically significant.

## Results

### ACTN4 Expression Increases During Androgen-Dependent PCa to AIPC Transition

**FIGURE 1:**
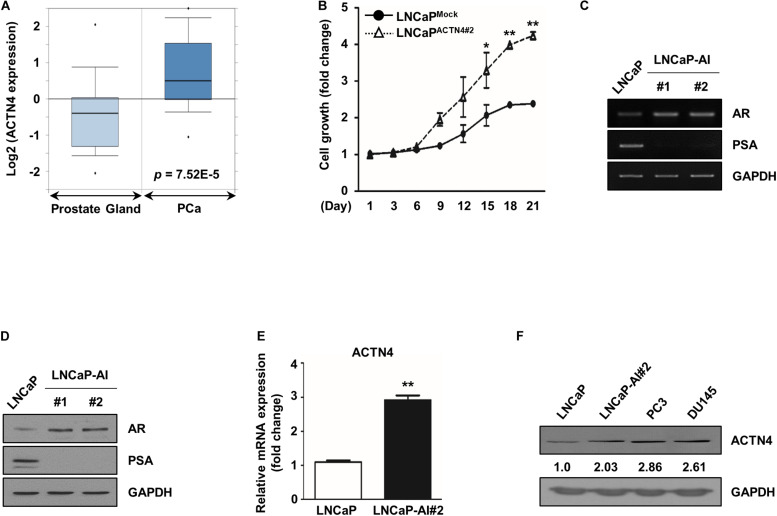
ACTN4 expression increases during androgen-dependent PCa to AIPC transition. **(A)** The dataset was accessed using the Oncomine database for Tomlins Prostate Statistics (GSE6099). Box plots depict the distribution of ACTN4 expression within each group (*p* = 7.52E–5). **(B)** LNCaP^Mock^ indicates the mock vector-expressing stable LNCaP cell line. LNCaP^ACTN4^ indicates the ACTN4-overexpressing stable LNCaP cell line. Among the two independent clones of LNCaP^ACTN4^ cells numbered as #1 and #2, the LNCaP^ACTN4#2^ cell line was used in the study. Proliferation of LNCaP^Mock^ and LNCaP^ACTN4#2^ cells in CSS media was measured using the MTT assay. Cells were treated with water-soluble tetrazolium salt solution and incubated at 37°C for 30 min, and the absorbance was measured at 450 nm. **(C)** LNCaP and LNCaP-AI cell lysates (4 × 10^5^ cells/well) were analyzed by semi-qRT-PCR. **(D)** LNCaP and LNCaP-AI cell lysates (4 × 10^5^ cells/well) were analyzed by western blotting. **(E)** ACTN4 mRNA levels were analyzed using qRT-PCR. **(F)** Total cell lysates were obtained from various PCa cells. The protein levels were determined by western blotting. All experiments were repeated at least thrice independently. *p < 0.05, **p < 0.01. Error bar, SEM (unpaired, two-tailed Student’s *t-*test).

ACTN4 contributes to aggressiveness and metastasis of various cancers (Honda, 2015); therefore, we investigated whether ACTN4 plays a role in the progression of PCa. We first analyzed ACTN4 expression in the PCa patient database. According to the Oncomine database for Tomlins Prostate Statistics (GSE6099), ACTN4 expression is elevated in prostate carcinoma compared to that in the prostate gland (Figure 1A). CRPC recurs through the reactivation of the AR signaling after androgen deprivation therapy (Katsogiannou et al., 2015). Interaction between ACTN4 and AR was predicted by protein–protein binding analysis (Capaia et al., 2018); therefore, we examined whether ACTN4 is involved in the regulation of androgen-dependent PCa to AIPC transition. To address this question, we generated ACTN4 overexpressing LNCaP cells (LNCaP^ACTN4^); the cells were cultured in CSS media to examine the resistance against androgen deprivation conditions. Results of the MTT assay revealed that proliferation of LNCaP^*ACTN*4#2^ cells was better than that of LNCaP^Mock^ cells in CSS media (Figure 1B). We also generated androgen-independent LNCaP cells (LNCaP-AI) from androgen-dependent LNCaP cells. LNCaP-AI cells exhibited higher AR expression and lower prostate specific antigen (PSA) expression at both mRNA and protein levels, compared with those in LNCaP cells (Figures 1C,D). The high AR and low PSA levels are well-known AIPC transition markers; therefore, we regarded LNCaP-AI cells as AIPC cells in following studies. The mRNA level of ACTN4 was threefold higher in LNCaP-AI cells compared to LNCaP cells (Figure 1E). In addition, the protein level of ACTN4 was twofold higher in LNCaP-AI cells compared to LNCaP cells (Figure 1F). We also observed the higher expression of ACTN4 in two AIPC cell lines, PC3 and DU145, compared with LNCaP cells (Figure 1F). These results indicate that ACTN4 expression increases during the transition from androgen-dependent PCa to AIPC and is involved in PCa survival under androgen deprivation.

### ACTN4 Promotes the Proliferation of PCa Cells in an Androgen Deprivation State

**FIGURE 2:**
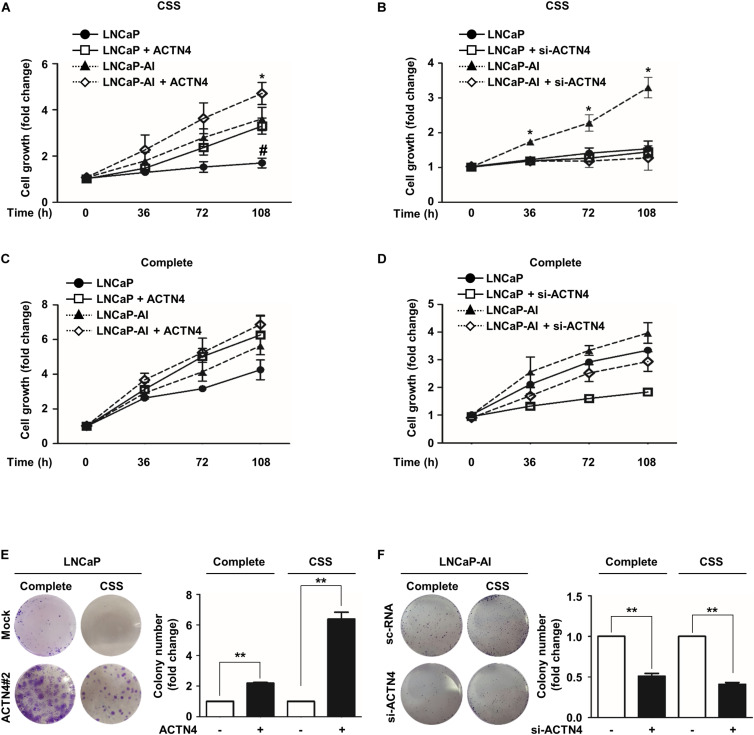
ACTN4 promotes the proliferation of PCa cells in an androgen deprivation state. **(A)** LNCaP and LNCaP-AI cells were transfected with Flag-ACTN4 (1 μg), and the cells (2 × 10^3^ cells/well) were cultured in CSS media (* is labeled as compared to LNCaP-AI cells, # is labeled as compared to LNCaP cells). **(B)** LNCaP and LNCaP-AI cells were transfected with si-ACTN4 (100 pmol/μl), and the cells (2 × 10^3^ cells/well) were cultured in CSS media. **(C,D)** LNCaP and LNCaP-AI cells (2 × 10^3^ cells/well) were transfected with Flag-ACTN4 (1 μg) or si-ACTN4 (100 pmol/μl) in complete media. Cell proliferation was measured using the MTT assay. Cells were treated with water-soluble tetrazolium salt solution and incubated for 30 min at 37°C and the absorbance was measured at 450 nm. **(E,F)** LNCaP^Mock^, LNCaP^ACTN4#2^, and LNCaP-AI cells (3 × 10^3^/well) were analyzed using a colony formation assay in complete or CSS media for 10 days. Colonies were stained with 0.05% crystal violet, and the colonies in at least five fields were counted. All experiments were repeated at least thrice independently. *p < 0.05, **p < 0.01, ^#^*p* < 0.05. Error bar, SEM (unpaired, two-tailed Student’s *t-*test).

ACTN4 is closely associated with malignancy and cell survival in many cancers (Honda, 2015; An et al., 2016). To examine whether ACTN4 affected PCa cell proliferation under androgen deprivation, we performed the MTT and colony formation assays. We first determined the level of si-ACTN4 used in this study in PC3 cells. ACTN4 knockdown decreased both the mRNA and protein levels of ACTN4 by 4- and 2.4-fold, respectively (Supplementary Figures S1A,B). ACTN4 overexpression rescued the knockdown of ACTN4 at both the mRNA and protein levels (Supplementary Figures S1A,B). Results of the MTT assay showed that ectopically expressed ACTN4 induced cell proliferation of androgen-dependent LNCaP cells under androgen deprivation conditions (Figure 2A). Moreover, ACTN4 enhanced the proliferation of LNCaP-AI cells in an androgen deprivation state, whereas ACTN4 knockdown suppressed the proliferation of LNCaP-AI cells (Figures 2A,B). In complete media, ACTN4 increased proliferation of LNCaP and LNCaP-AI cells, and ACTN4 knockdown decreased the cell proliferation (Figures 2C,D). Results of the colony formation assay showed that ACTN4 increased the proliferation of LNCaP cells by 2.1- and 6.3-fold in complete and CSS media, respectively (Figure 2E). ACTN4 knockdown decreased the proliferation of LNCaP-AI cells by twofold in both complete and CSS media (Figure 2F). These results indicate that ACTN4 promotes proliferation of PCa cells in an androgen deprivation state.

### ACTN4 Induces the Migration and Invasion of PCa Cells

**FIGURE 3:**
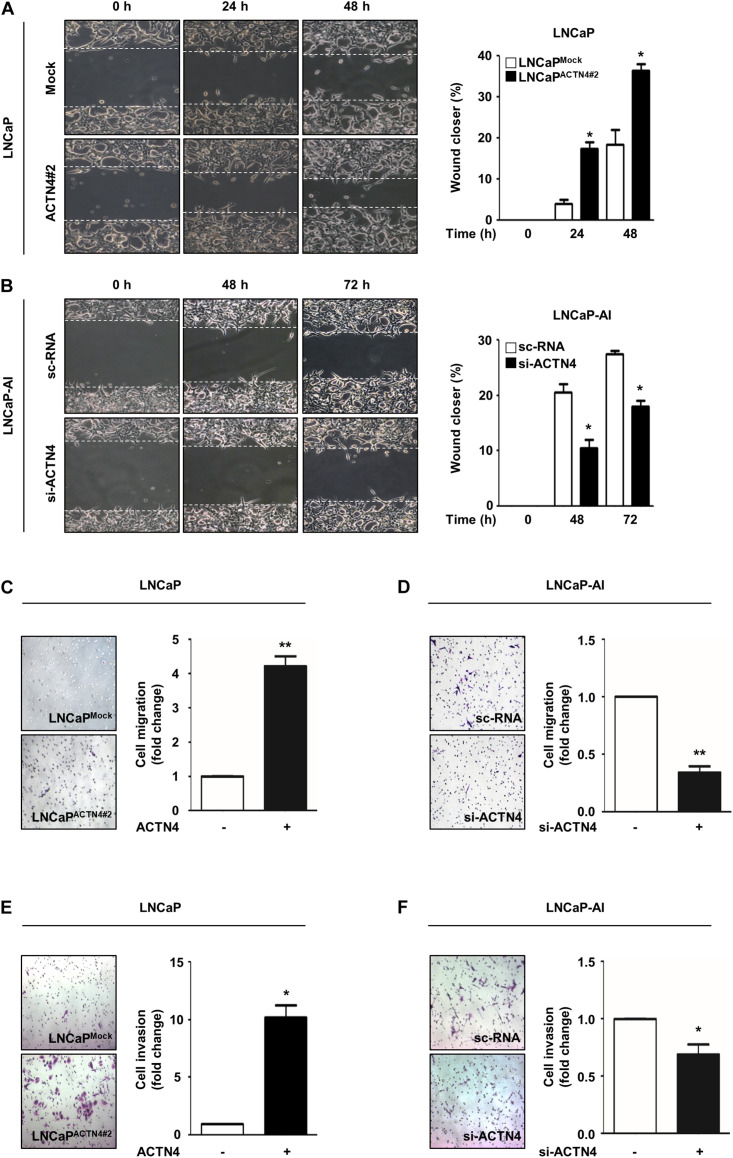
ACTN4 induces the migration and invasion of PCa cells. **(A)** Cell culture plates with attached LNCaP cells were wounded and incubated with serum free RPMI 1640 medium. Representative wound-healing images were taken 0–48 h after wound scratch. **(B)** LNCaP-AI cells were transfected with sc-RNA or si-ACTN4 (100 pmol/μl) for 48 h and cultured in CSS media. Wound healing was monitored for 0–72 h. **(C,D)** LNCaP^Mock^, LNCaP^ACTN4#2^, or si-ACTN4-transfected LNCaP-AI cells (1 × 10^5^ cells/well) were incubated for 48 h. Transwell migration was determined using 0.05% crystal violet staining. Cells from at least five fields were counted with the exception of non-specific stains. **(E,F)** For the transwell invasion assay, LNCaP^Mock^ and LNCaP^ACTN4#2^, or si-ACTN4-transfected LNCaP-AI cells (1.5 × 10^5^ cells/well) were stained with 0.05% crystal violet solution after 72 h incubation. All experiments were repeated thrice independently. *p < 0.05, **p < 0.01. Error bar, SEM (unpaired, two-tailed Student’s *t*-test).

ACTN4 induces the EMT and promotes cell migration and invasion in cervical cancer (An et al., 2016). To investigate whether ACTN4 was involved in the metastatic properties of PCa cells, we examined the effects of ACTN4 on migration and invasion of PCa cells. Results of the wound healing assay showed that compared with LNCaP^Mock^ cells, LNCaP^ACTN4#2^ cells showed an increased wound closing rate in a time-dependent manner (Figure 3A). Furthermore, compared with the scrambled si-RNA (sc-RNA), si-ACTN4 reduced the wound closing rate in LNCaP-AI cells (Figure 3B). We also performed a transwell migration assay in LNCaP and LNCaP-AI cells. Compared with the LNCaP^Mock^ cells, LNCaP^ACTN4#2^ cells showed 4.2-fold higher migration (Figure 3C). However, compared with the sc-RNA, si-ACTN4 reduced cell migration by 3.3-fold in LNCaP-AI cells (Figure 3D). In addition, LNCaP^ACTN4#2^ cells exhibited an increased cell invasion by 10.2-fold compared with LNCaP^Mock^ cells (Figure 3E). However, compared with the control, ACTN4 knockdown decreased the invasion of LNCaP-AI cells by 1.6-fold (Figure 3F). The specificity of si-ACTN4 in cell migration was determined in PC3 cells. ACTN4 knockdown reduced cell migration by 3.6-fold, whereas the rescued ACTN4 restored cell migration, indicating ACTN4 knockdown was specific (Supplementary Figure S2A). These results indicate that ACTN4 induces the migration and invasion of PCa cells.

### ACTN4 Induces β-Catenin Accumulation in the Nucleus by Activating Akt and GSK-3β

**FIGURE 4:**
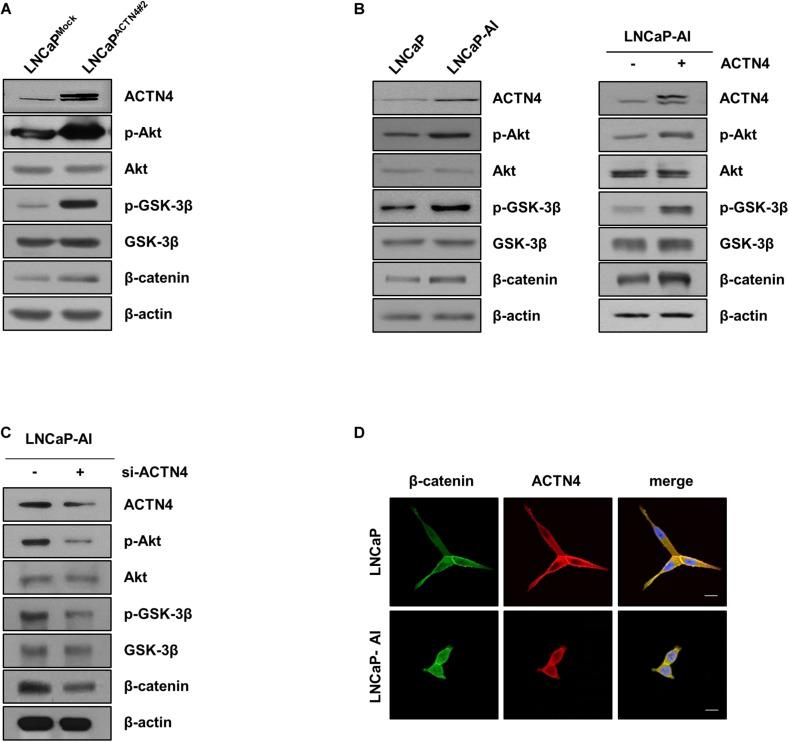
ACTN4 induces β-catenin accumulation in the nucleus by activating Akt and GSK-3β. **(A)** LNCaP^Mock^ and LNCaP^ACTN4#2^ cells (4 × 10^5^ cells/well) were harvested and the protein levels were detected by western blotting using the indicated antibodies. **(B)** Western blotting was performed using whole lysates of LNCaP and LNCaP-AI cells (4 × 10^5^ cells/well). LNCaP-AI cells (4 × 10^5^ cells/well) were transfected with Flag-ACTN4 (1 μg) for 24 h. Cell lysates were electrophoresed on an 8% SDS-PAGE and the protein levels were determined by western blotting. **(C)** LNCaP-AI cells (4 × 10^5^ cells/well) were transfected with sc-RNA or si-ACTN4 (100 pmol/μl) for 48 h, and the protein levels were determined by western blotting. **(D)** LNCaP and LNCaP-AI cells were incubated with anti-β-catenin and anti-ACTN4 antibodies. Cells were washed and incubated with secondary antibodies conjugated to Alexa 488 (green) and Alexa 594 (red). The nucleus (blue) was stained with DAPI. Scale bar, 20 μm.

Our previous study suggests that ACTN4 is involved in Akt-mediated β-catenin transcriptional activation in cervical cancer (An et al., 2016). Since the β-catenin signaling pathway is critical for tumor growth and metastasis, we examined whether ACTN4 influences the β-catenin activation in PCa. Ectopically expressed ACTN4 activated the Akt/GSK-3β/β-catenin signaling pathway in LNCaP cells (Figure 4A). High expression of ACTN4 in LNCaP-AI cells increased the phosphorylation of Akt and GSK-3β (Figure 4B). It also induced the stabilization of β-catenin (Figure 4B). We also examined the effect of ACTN4 overexpression on the Akt/GSK-3β/β-catenin signaling in LNCaP-AI cells. Results showed that ACTN4 overexpression activated the Akt/GSK-3β/β-catenin signaling in LNCAP-AI cells (Figure 4B). ACTN4 knockdown reduced the Akt-mediated β-catenin stabilization (Figure 4C). We examined the localization of β-catenin in LNCaP and LNCaP-AI cells. The results of immunofluorescence staining showed that β-catenin was apparently accumulated in the nucleus in LNCaP-AI cells compared to LNCaP cells (Figure 4D). These results indicate that ACTN4 induces β-catenin accumulation in the nucleus by activating Akt and GSK-3β.

### ACTN4 Induces the Expression of Cell Proliferation and EMT Markers Through the Akt/GSK-3β/β-Catenin Signaling Pathway

**FIGURE 5:**
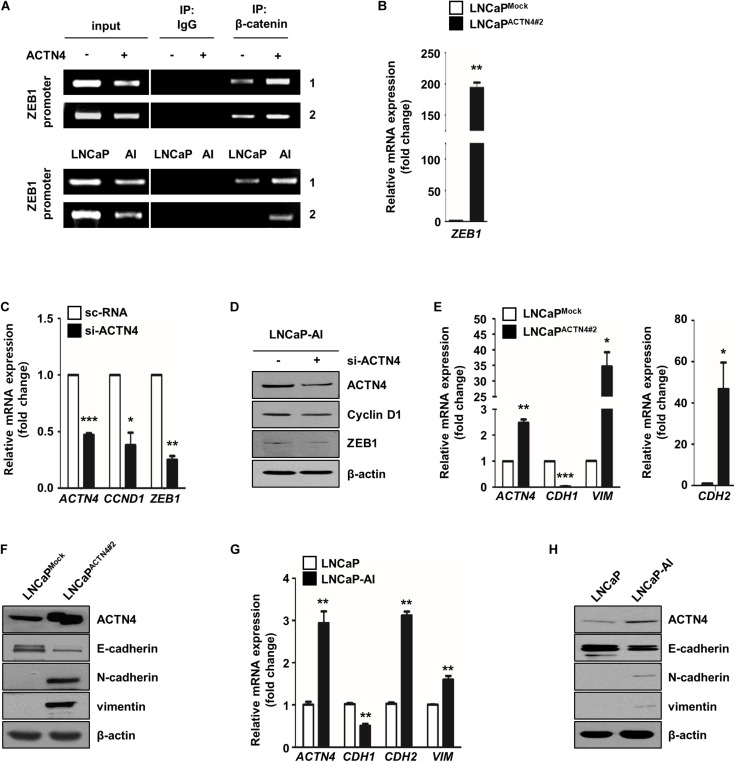
ACTN4 induces the expression of cell proliferation and EMT markers through the Akt/GSK-3β/β-catenin signaling pathway. **(A)** The DNA–protein complexes were precipitated using anti-β-catenin antibody. Purified DNA fragments were confirmed by semi-qRT-PCR using the TCF/LEF specific primers (1; −674 to −477 bp and 2; −325 to −101 bp) in the ZEB1 promoter. IgG was used as a negative control. **(B)** The ZEB1 mRNA level was analyzed by qRT-PCR. **(C,D)** qRT-PCR was performed to determine the mRNA expression, and the protein levels were determined by western blotting. **(E,F)** qRT-PCR and western blotting were performed to determine the EMT marker expression. **(G)** Total lysates were obtained from LNCaP and LNCaP-AI cells. The mRNA levels were determined by qRT-PCR. **(H)** The protein levels of LNCaP and LNCaP-AI cells were determined by western blotting using the indicated EMT marker antibodies. All experiments were repeated at least thrice independently *p < 0.05, **p < 0.01, ***p < 0.001. Error bar, SEM (unpaired, two-tailed Student’s *t*-test).

To investigate the regulation of PCa cell proliferation and EMT by ACTN4, we examined the effect of ACTN4 on the expression of β-catenin targets such as cyclin D1 and ZEB1. ZEB1 promoter has two β-catenin binding sites (Sanchez-Tillo et al., 2011). Therefore, we examined whether ACTN4 regulates the binding of β-catenin to ZEB1 promoter. We performed a ChIP assay in LNCaP and LNCaP-AI cells. LNCaP^ACTN4#2^ cells showed an enhanced β-catenin binding to the ZEB1 promoter compared to LNCaP^Mock^ cells (Figure 5A). LNCaP-AI cells, which show a high expression of ACTN4, also showed an increased β-catenin binding to ZEB1 promoter compared to that in LNCaP cells (Figure 5A). When we examined the effect of ACTN4 on ZEB1 transcription, ACTN4 increased the ZEB1 mRNA expression by 200-fold in LNCaP cells (Figure 5B). These results indicate that ACTN4 increases the ZEB1 transcription by enhancing the β-catenin binding to the ZEB1 promoter. In addition, ACTN4 knockdown suppressed the mRNA and protein expression of cyclin D1 and ZEB1 (Figures 5C,D). ZEB1 functions as a key regulator in EMT (Gillett et al., 1996; Khan et al., 2015); therefore, we examined the effect of ACTN4 on the expression of EMT makers. ACTN4 decreased E-cadherin expression, whereas increased vimentin and N-cadherin mRNA and protein expressions in LNCaP cells (Figures 5E,F). LNCaP-AI cells exhibited a decrease in E-cadherin expression compared to LNCaP cells (Figures 5G,H). However, compared with LNCaP cells, LNCaP-AI cells showed higher expression of vimentin and N-cadherin at the mRNA and protein levels (Figures 5G,H). These results indicate that ACTN4 regulates cell proliferation and EMT through the Akt/GSK-3β/β-catenin signaling pathway.

### ACTN4 Promotes the Motility of AIPC Cells and Castration-Resistant Tumor Growth *in vivo*

**FIGURE 6:**
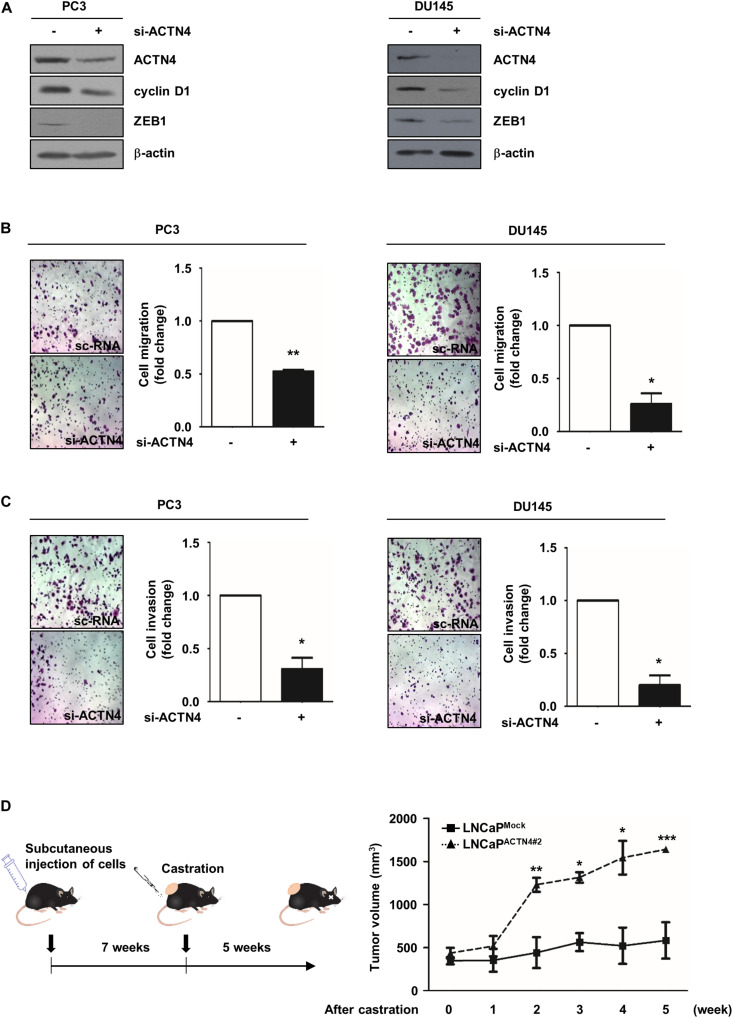
ACTN4 promotes the motility of AIPC cells and castration-resistant tumor growth *in vivo*. **(A)** PC3 and DU145 cells (4 × 10^5^ cells/well) were transfected with sc-RNA or si-ACTN4 (100 pmol/μl) for 48 h, and the cell lysates were analyzed by western blotting. **(B,C)** PC3 and DU145 cells were transfected with sc-RNA or si-ACTN4 (100 pmol/μl) for 48 h, and the cells were incubated for 24 h. For migration and invasion assays, the cells were stained using 0.05% crystal violet, and those in at least five fields were counted. **(D)** Nude mice were subcutaneously injected with LNCaP^Mock^ (4 × 10^6^ cells/well) and LNCaP^ACTN4#2^ (3 × 10^6^ cells/well) cells, and mice with tumor were castrated. Mice were killed 5 weeks after castration (*n* = 3). Tumor volume was calculated according to the following equation: *V* = 0.5 × (width^2^ × length). All experiments were repeated thrice independently. *p < 0.05, **p < 0.01, ***p < 0.001. Error bar, SEM (unpaired, two-tailed Student’s *t*-test).

We next investigated the effect of ACTN4 on the progression of AIPC using AIPC cell lines, PC3, and DU145. ACTN4 knockdown decreased cyclin D1 and ZEB1 expression in PC3 and DU145 cells (Figure 6A). To examine the effect of ACTN4 on cell motility and invasiveness, we performed migration and invasion assays in PC3 and DU145 cells. ACTN4 knockdown reduced cell migration by 1.9- and 3.8-fold in PC3 and DU145 cells, respectively (Figure 6B). Results of the invasion assay showed that compared to the sc-RNA, si-ACTN4 reduced invasiveness by 3.2- and 5-fold in PC3 and DU145 cells, respectively (Figure 6C). These results indicate that ACTN4 promotes the motility of AIPC cells. To verify the role of ACTN4 in PCa progression after castration, we performed xenograft experiments using nude mice. LNCaP^Mock^ and LNCaP^ACTN4#2^ cells were subcutaneously injected into nude mice. Testicular hormone was eliminated by castration when the tumor size reached approximately 400 mm^3^. Tumors in the mice injected with LNCaP^ACTN4#2^ cells continued to grow after castration (Figure 6D). However, tumors in the mice injected with LNCaP^*Mock*^ cells did not grow after castration (Figure 6D). These results indicate that ACTN4 is required for the acquisition of castration-resistance property in PCa.

### Anti-ACTN4 Nanobody Inhibits the Proliferation and Motility of PCa Cells

**FIGURE 7:**
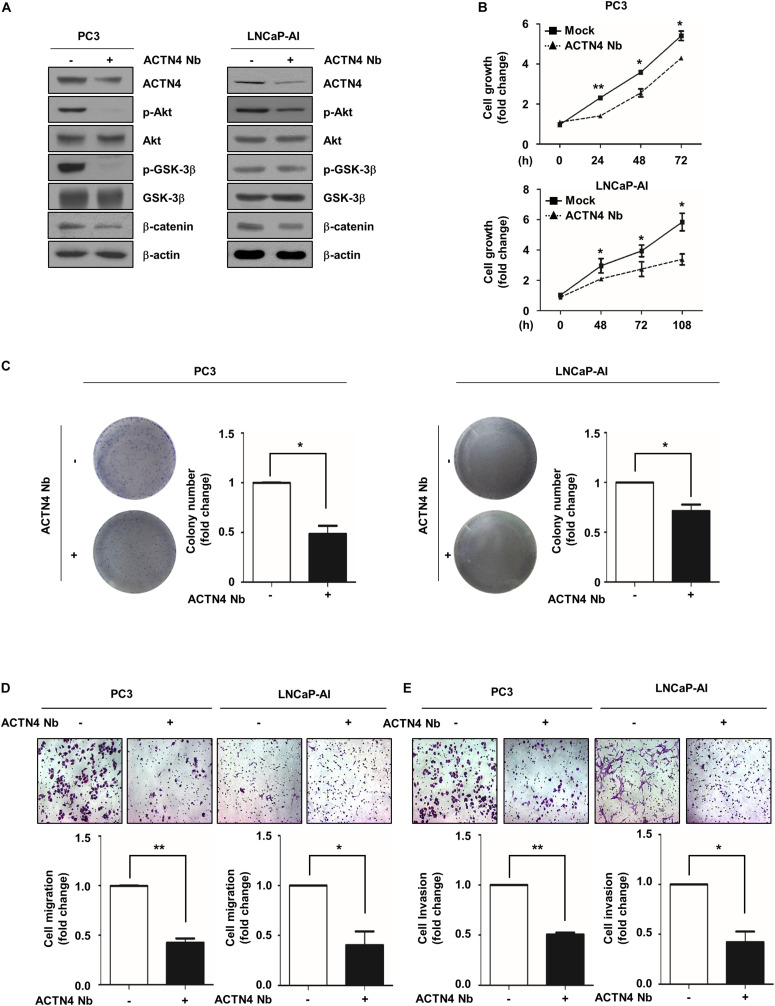
ACTN4 Nb inhibits the proliferation and motility of AIPC cells. **(A)** PC3 and LNCaP-AI cells (4.5 × 10^5^ cells/well) were transfected with Flag-Mock and Flag-ACTN4 Nb (1 μg) for 24 h, and cells lysates were analyzed by western blotting. **(B)** PC3 (1 × 10^4^ cells/well) and LNCaP-AI (5 × 10^3^ cells/well) cells were transfected with Flag-Mock and Flag-ACTN4 Nb (1 μg). Cell proliferation was determined by the MTT assay. Cells were treated with water-soluble tetrazolium salt solution and incubated for 30 min at 37°C, and the absorbance was measured at 450 nm. **(C)** PC3 and LNCaP-AI cells (3 × 10^3^ cells/well) were analyzed by a colony formation assay for 3 and 7 days. Colonies were stained with 0.05% crystal violet, and the colonies in at least five fields were counted. **(D,E)** For the transwell migration and invasion assays, PC3 cells transfected with ACTN4 Nb (5 × 10^4^ cells/well) were incubated for 24 h, and LNCaP-AI cells transfected with ACTN4 Nb (1 × 10^5^ cells/well) were incubated for 72 h. The cells were stained using 0.05% crystal violet, and those in at least five fields were counted. All experiments were repeated thrice independently. *p < 0.05, **p < 0.01. Error bar, SEM (unpaired, two-tailed Student’s *t*-test).

**FIGURE 8:**
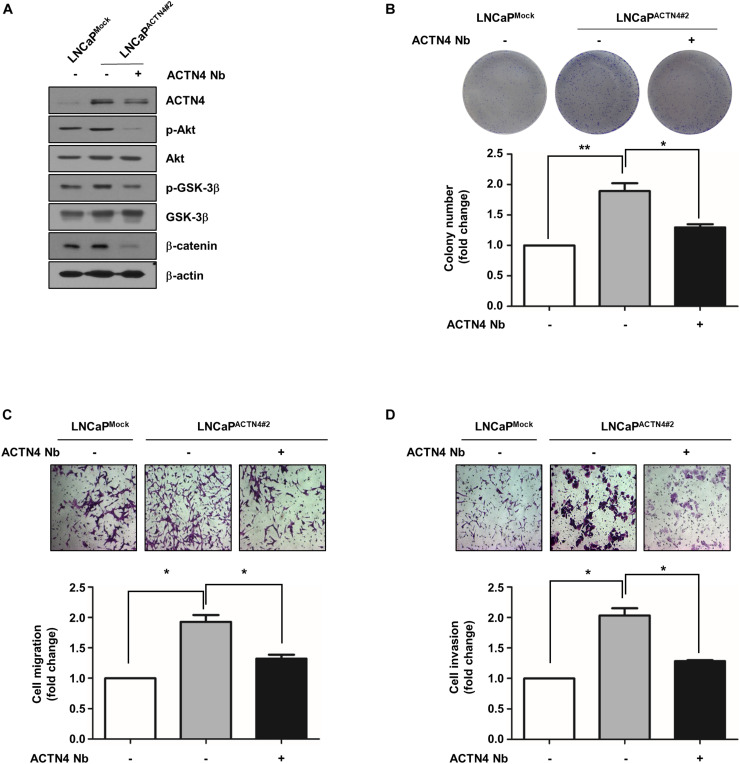
ACTN4 Nb inhibits the proliferation and motility of androgen-dependent PCa cells. **(A)** Cell proliferation was analyzed by a colony formation assay. LNCaP^Mock^ and LNCaP^ACTN4#2^ cells (4.5 × 10^5^ cells/well) were transfected with Flag-Mock and Flag-ACTN4 Nb (1 μg) for 24 h. Total cell lysates were analyzed by western blotting using the indicated antibodies. **(B)** For colony formation assay, LNCaP^Mock^ and LNCaP^ACTN4#2^ cells (3 × 10^3^ cells/well) were incubated for 7 days. The cells were stained with 0.05% crystal violet and then counted. **(C,D)** LNCaP^Mock^ and LNCaP^ACTN4#2^ cells (4.5 × 10^5^ cells/well) were transfected with Flag-Mock and Flag-ACTN4 Nb (1 μg) for 24 h. The cells (1 × 10^5^ cells/well) were analyzed by transwell migration and invasion assays. The cells were stained using 0.05% crystal violet, and those in at least five fields were counted. All experiments were repeated thrice independently. *p < 0.05, **p < 0.01. Error bar, SEM (unpaired, two-tailed Student’s *t*-test).

Nanobodies (Nbs) were developed to successfully target and inhibit the human chemokine receptors and targeted treatment of tumors (Hu et al., 2017). Anti-ACTN4 Nb (Nb64) was developed by Gulliver Biomed BV (Ghent, Belgium) through the immunization of llamas. To investigate the effect of Nb64 on the progression of PCa, we generated the Flag-ACTN4 Nb64 construct (ACTN4 Nb) and transfected it to PCa cells. ACTN4 Nb was cloned into pCMV-3tag-1 vector using *Apa*I and *Eco*RI restriction enzymes. ACTN4 Nb inhibited the Akt/GSK3β/β-catenin signaling pathway in both PC3 and LNCaP-AI cells (Figure 7A). Results of MTT assay showed that ACTN4 Nb suppressed the growth of PCa cells in both cell lines (Figure 7B). We found that ACTN4 Nb decreased the colony formation of both PC3 and LNCaP-AI cells approximately by twofold (Figure 7C). We examined the effects of ACTN4 Nb on the migration and invasion of PC3 and LNCaP-AI cells. ACTN4 Nb reduced cell migration by 2.6- and 2.4-fold in PC3 and LNCaP-AI cells, respectively (Figure 7D). ACTN4 Nb also reduced cell invasion by 1.9- and 2.3-fold in PC3 and LNCaP-AI cells, respectively (Figure 7E). These results indicate that ACTN4 Nb is specifically targeted at ACTN4 and inhibits the proliferation and motility of PCa cells. We also examined the effects of ACTN4 Nb on the proliferation and motility of LNCaP^ACTN4^ cells. ACTN4 Nb efficiently suppressed the Akt/GSK-3β/β-catenin signaling despite the overexpression of ACTN4 (Figure 8A). Results of colony formation assay showed that LNCaP^ACTN4#2^ cells exhibited a twofold increase in colony formation compared to LNCaP^Mock^ cells; however, ACTN4 Nb decreased the colony formation of LNCaP^ACTN4#2^ cells by 1.3-fold (Figure 8B). In addition, ACTN4 Nb reduced the migration and invasion of LNCaP^ACTN4#2^ cells (Figures 8C,D). These results suggest that ACTN4 Nb inhibits the proliferation and motility of PCa cells.

## Discussion

ACTN4 functions as a coactivator of oncogenic transcription factors in cervical, lung, colorectal, and breast cancers (Menez et al., 2004; Khurana et al., 2011; Fukumoto et al., 2015). ACTN4 mutation causes the suppression of tumor cell growth and migration in lung carcinoma (Menez et al., 2004). ACTN4 also increases cell motility and promotes lymph node metastasis in colorectal cancer (Fukumoto et al., 2015). In addition, ACTN4 knockdown reduces ERα target gene expression and decreases the proliferation of breast cancer cells (Khurana et al., 2011). In this study, we investigated the role of ACTN4 in the progression of PCa. Especially, we focused on the role of ACTN4 in the transition of androgen-dependent PCa to AIPC. We observed that ACTN4 expression was elevated in LNCaP-AI cells compared to that in LNCaP cells. Interestingly, LNCaP^ACTN4#2^ cells maintained a steady cell proliferation rate in CSS media, whereas proliferation of LNCaP^*Mock*^ cells was decreased, indicating that ACTN4 is involved in androgen-independent proliferation of PCa cells. ACTN4 knockdown decreases the activation of Akt and GSK-3β, and it reduces cell motility in breast cancer (Desai et al., 2018). shACTN4 suppresses the Akt/GSK-3β/β-catenin signaling, and ACTN4 interacts with β-catenin in breast cancer stem cells (Wang et al., 2017). In addition, the ACTN4 and TRIP13 complex promotes EMT and tumor metastasis through the Akt signaling in hepatocellular carcinoma cells (Zhu et al., 2019). We have previously reported that ACTN4 induces the phosphorylation of Akt and GSK-3β in cervical cancer (An et al., 2016). We found that ACTN4 promotes cell motility and proliferation through the Akt/GSK3β/β-catenin pathway in PCa.

CRPC occurs due to relapse after androgen deprivation therapy (Sun et al., 2012). Castration and androgen deprivation reinforce the EMT properties, and EMT causes PCa metastasis and CRPC development (Li et al., 2014). According to reports, the expression of EMT-related factors such as N-cadherin, ZEB1, Twist1, and Slug at the transcriptional level is increased when mice are castrated (Sun et al., 2012). We found that ACTN4 induced N-cadherin, ZEB1, and vimentin in LNCaP-AI and LNCaP^ACTN4#2^ cells. We also found that ACTN4 enhanced the binding of β-catenin to the ZEB1 promoter in AIPC cells. These results demonstrate that ACTN4 is involved in the progression of PCa by promoting cell motility and EMT in androgen-dependent PCa and AIPC cells.

The glucocorticoid receptor (GR) is closely related to AR and can functionally replace AR when AR signaling is blocked (Arora et al., 2013). GR expression is reduced in primary PCa tissues compared to benign tissues; however, restored GR expression during the CRPC transition is critical for CRPC cell proliferation (Puhr et al., 2018). Heme oxygenase 1 reduces cell proliferation by the inhibition of GR in PC3 cells (Leonardi et al., 2019). Furthermore, replacement of reduced AR signaling with GR activation increases cell survival by inducing anti-apoptotic serum/glucocorticoid-regulated kinase 1 (Isikbay et al., 2014). Since ACTN4 interacts with GR and participates in GR activation (Zhao et al., 2017), ACTN4 may be a key regulatory factor for PCa recurrence after androgen deprivation therapy.

Recent studies have reported that Na^+^/H^+^ exchanger regulatory factor 1 (NHERF1) is associated with cell proliferation through the Wnt/β-catenin signaling and negatively regulates ACTN4 expression in cervical cancer (Wang et al., 2018). In the Oncomine database (GSE6099), NHERF1 mRNA expression is reduced in prostate carcinoma compared to PCa precursor. Therefore, NHERF1 may be an upstream regulator of ACTN4. In addition, LIM domain kinase 1 (LIMK1), which is overexpressed in colorectal cancer, promotes cell motility and proliferation via Akt signaling, and ACTN4 is a downstream target gene of LIMK1 in colorectal cancer (Liao et al., 2017). However, the regulatory mechanism of ACTN4 in the progression of PCa is poorly understood. Although further studies are needed to characterize the molecular mechanism of CRPC transition and PCa progression, our findings suggest that ACTN4 is involved in advancement of the EMT potential of PCa cells and acquisition of the aggressive cancer properties. Therefore, ACTN4 can be a therapeutic target to prevent tumor survival, metastasis, and recurrence of PCa.

Monoclonal antibodies have been used for the development of tumor-targeted therapies (Kijanka et al., 2015). These antibodies cause less damage to healthy cells around the tumor site and have been used to directly inhibit tumor growth and proliferation or to deliver effector molecules to tumor cells (Bhutani and Vaishampayan, 2013). However, the size of monoclonal antibodies is about 150 kDa, preventing their access to tumors (Hu et al., 2017). To overcome these limitations, smaller formats such as Nbs have been generated (Bertier et al., 2017; Beghein et al., 2018). Nbs are small in size, less than 15 kDa, and have the advantage of being able to easily penetrate tumors through blood vessels in the body and persist in the tumor for a long time (Roovers et al., 2011). Recent studies have shown that several tumors are treated with Nbs. Angiogenesis is an important process for the growth of solid tumors, and many Nbs have been generated to interfere with vascularization. Vascular endothelial growth factor receptor 2, a factor that induces angiogenesis, is overexpressed in many types of cancers, including lung and colon cancers (Olsson et al., 2006). The 3VGR19 Nb targeting vascular endothelial growth factor receptor 2 inhibits capillary tube formation *in vitro* (Behdani et al., 2012). In addition, the 5F7GGC Nb targeting HER2, which plays an important role in inducing the growth, survival, and differentiation of breast cancer cells, binds to tumor cells and effectively inhibits the growth of tumors (Pruszynski et al., 2013). We showed that ACTN4 Nb reduces the growth and motility of PCa cells. Although further studies are required to determine the effect of ACTN4 Nb on the progression of PCa *in vivo*, our results suggest that ACTN4 Nb can be a therapeutic molecule for the treatment of PCa.

## Data Availability Statement

The original contributions presented in the study are included in the article/Supplementary Material, further inquiries can be directed to the corresponding author/s.

## Ethics Statement

The animal study was reviewed and approved by The Institutional Animal Care and Use Committee of Korea University.

## Author Contributions

SP and JK participated in conception and design of the experiments, contributed to data analysis and interpretation, and wrote the manuscript. MK and H-TA contributed to design of the experiments and data analysis. SP and SK performed experiments. JG provided the materials. All authors contributed to the article and approved the submitted version.

## Conflict of Interest

The authors declare that the research was conducted in the absence of any commercial or financial relationships that could be construed as a potential conflict of interest.
