# Supplementary material for: α-Actinin-4 Promotes the Progression of Prostate Cancer Through the Akt/GSK-3β/β-Catenin Signaling Pathway
Source: Front Cell Dev Biol. 2020 Dec 10;8:588544. doi: 10.3389/fcell.2020.588544 (PMC7758325; doi:10.3389/fcell.2020.588544)
Supplement: Supplementary file 1 [file Data_Sheet_1.docx]

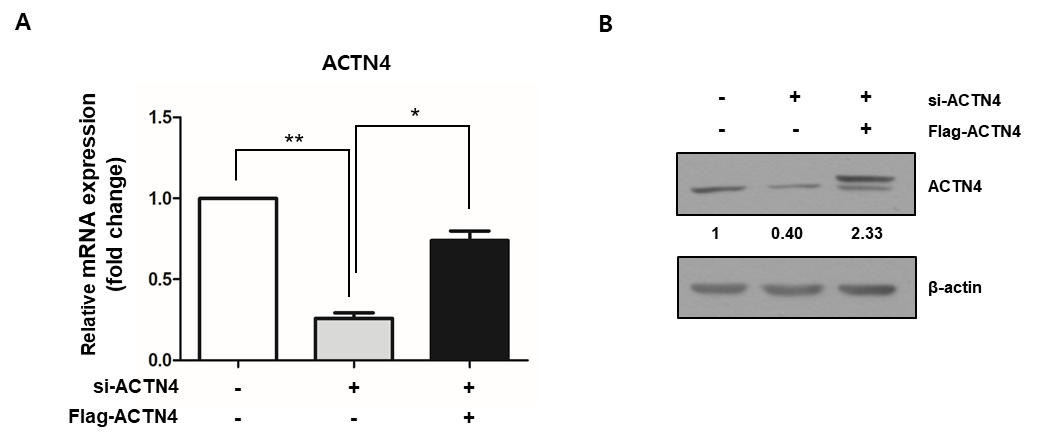


**Supplemental Figure S1.** (A and B) PC3 cells (4 × 10^5^ cells/well) were transfected with si-ACTN4 (100 pmol/μl) for 48 h and then with Flag-ACTN4 (1 μg) for 24 h. (A) Cell lysates were analyzed by qRT-PCR. All experiments were repeated at least thrice independently. (B) Cell lysates were electrophoresed on an 8% SDS-PAGE and the protein levels were determined by western blotting. All experiments were repeated at least thrice independently. **p* < 0.05, ***p* < 0.01. Error bar, SEM (unpaired, two-tailed Student’s t-test).


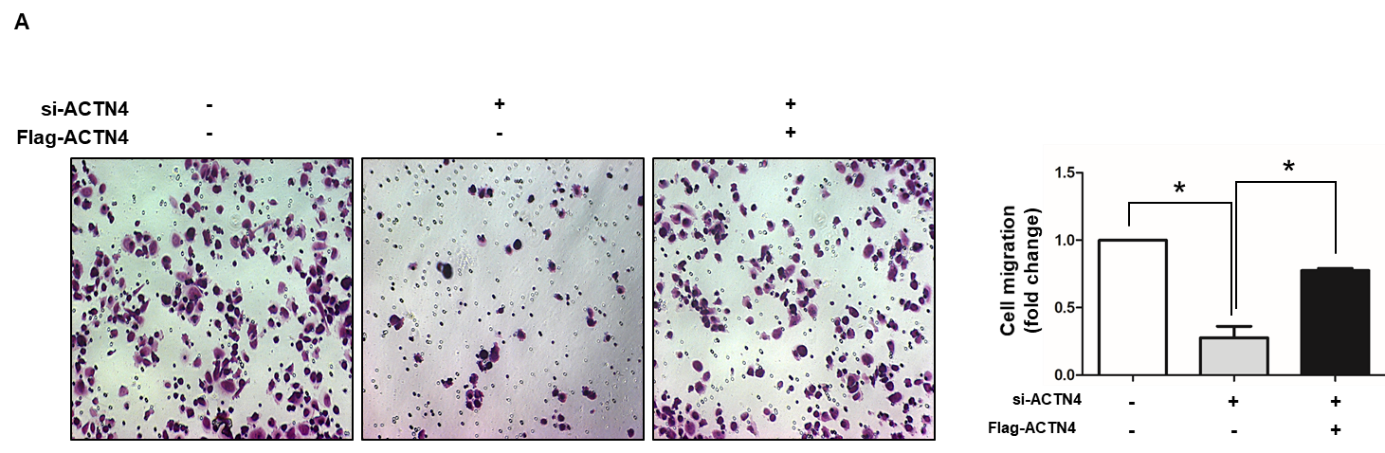


**Supplemental Figure S2.** (A) PC3 cells (4 × 10^5^ cells/well) were transfected with si-ACTN4 (100 pmol/μl) for 48 h and then with Flag-ACTN4 (1 μg) for 24 h. For the transwell migration assay, transfected PC3 cells (1 × 10^5^ cells/well) were stained with 0.05% crystal violet solution after 24 h incubation. All experiments were repeated thrice independently. **p* < 0.05. Error bar, SEM (unpaired, two-tailed Student’s *t*-test).
